# Identifying Node Role in Social Network Based on Multiple Indicators

**DOI:** 10.1371/journal.pone.0103733

**Published:** 2014-08-04

**Authors:** Shaobin Huang, Tianyang Lv, Xizhe Zhang, Yange Yang, Weimin Zheng, Chao Wen

**Affiliations:** 1 College of Computer Science and Technology, Harbin Engineering University, Harbin, China; 2 College of Information Science and Engineering, Northeastern University, Shenyang, China; 3 College of Computer Science and Technology, Tsinghua University, Beijing, China; 4 Audit Research Institute, National Audit Office, Beijing, China; Semmelweis University, Hungary

## Abstract

It is a classic topic of social network analysis to evaluate the importance of nodes and identify the node that takes on the role of core or bridge in a network. Because a single indicator is not sufficient to analyze multiple characteristics of a node, it is a natural solution to apply multiple indicators that should be selected carefully. An intuitive idea is to select some indicators with weak correlations to efficiently assess different characteristics of a node. However, this paper shows that it is much better to select the indicators with strong correlations. Because indicator correlation is based on the statistical analysis of a large number of nodes, the particularity of an important node will be outlined if its indicator relationship doesn't comply with the statistical correlation. Therefore, the paper selects the multiple indicators including degree, ego-betweenness centrality and eigenvector centrality to evaluate the importance and the role of a node. The importance of a node is equal to the normalized sum of its three indicators. A candidate for core or bridge is selected from the great degree nodes or the nodes with great ego-betweenness centrality respectively. Then, the role of a candidate is determined according to the difference between its indicators' relationship with the statistical correlation of the overall network. Based on 18 real networks and 3 kinds of model networks, the experimental results show that the proposed methods perform quite well in evaluating the importance of nodes and in identifying the node role.

## Introduction

In social science, social network analysis (SNA) is the analysis of a social structure that is made up of a set of social actors and a set of the interactions between these actors. Individual such as human, or organization such as school, corporation and nation, can be considered to be a social actor [1]. In recent years, with the widespread use of social media such as *FaceBook* and *Twitter*, a vast amount of social interaction data has made social network analysis go beyond sociology and attract researchers from various fields.

The progress of social network analysis has also benefited from the researches on complex network. Since the late 20th century, after Watts D. J. and Barabasi A. L. successfully explained the phenomena of small-world and scale-free [2], [3], complex network has become the fundamental model to understand complex topological relations and dynamic behaviors in various fields [4], such as the Internet [5], epidemic spreading [6], etc. In these fields, evaluating the importance of nodes is of great value [14].

Social network analysis is concerned not only with evaluating the importance of a node, but also with identifying the function or position of an important node in a network. As *John Scott* stated in 1991, it has been one of the key issues of social network analysis to identify the role of a node [13].

Although various kinds of roles could be defined from different perspectives, two kinds are widely accepted [37], [38]. The first kind of role bonds a group of nodes together, and has great influence on other nodes. A node that plays the bonding role usually takes up the central position of the group, thus it is named as a *core* in this paper. In previous studies, this kind of role has also been termed as a *leader*, a *star*, a *hub* etc. The second kind of role provides connections between other nodes. A node that plays this role looks like a *bridge* and shows its importance in exchanging information and resources between others [7], [8], [9].

However, there are still many arguments about the precise definitions of the *core* and the *bridge*. Therefore, instead of pursuing the precise definition of a role [10], researchers have proposed many different indicators to assess different topological features of a node [11], [12], such as degree, betweenness centrality, eigenvector centrality etc. And researchers have usually agreed that the degree of a *core* and the betweenness centrality of a *bridge* should be great. However, any single indicator is not sufficient to identify multiple and complex characteristics of a role. For instance, a *core* is also an important part of information exchange particularly between the nodes bonded by the *core* itself.

A promising solution is to apply multiple indicators to evaluate the importance of a node and identify its role. However, the number of different indicator combinations increases exponentially with the number of indicators. For instance, there are 1024 different combinations of just 10 indicators. And the study of how to select the appropriate combination has not come to a conclusion yet [13]. An intuitive solution is to select the indicators with weak correlations. Its basic assumption is that the indicators with weak correlations could be good at assessing different topology features.

However, this solution is not appropriate for analyzing an individual node, although it may be suitable for a network or a set of nodes. Because the correlation between indicators reflects the statistical relationship of a large number of nodes, the particularity of an individual node would be outlined if its indicator correlation conflicts with the statistical relationship. Therefore, the indicators with strong correlations should be selected to highlight important individuals. This deduction is also confirmed by sociologists' preliminary analysis of a few indicators. For example, degree is usually positively correlated with closeness centrality, but if the two indicators of a node do not satisfy this relationship, the node should play an important role in connecting with some other important nodes.

Therefore, this paper proposes to select the multiple indicators with strong correlations to evaluate the importance of a node and identify its role in an undirected no-weighted social network. Besides the correlation between indicators, the paper also takes the range of application of an indicator, the topology feature evaluated by an indicator and the topological information required by an indicator into consideration in selecting desired multiple indicators. Eventually, the indicators, *degree*, *ego betweenness centrality* and *eigenvector centrality* are selected from 10 typical indicators of SNA and complex network analysis. Then, the importance of a node is equal to the normalized sum of its three indicators; the core candidates are selected from the nodes with great degree and the bridge candidates are selected from the nodes with great ego-betweenness centrality. Finally, the role of a candidate is determined according to the difference between its indicators' relationship with the statistical correlation of the overall network. If the node shows its significance in connecting non-adjacent nodes together and in connecting with other important nodes, the node is recognized as a core or a bridge. It is noteworthy that the selected indicators can also be computed based on the ego network of a node instead of based on the overall network. This feature makes the proposed method highly adaptable to the large, time-varying network whose precise and up-to-date global topology is hard to be obtained. The experimental results show the good performance of the proposed method, especially in analyzing the scale-free networks.

The rest part of this paper is arranged as follows: Section 2 analyzes the correlations of 10 typical indicators and shows the drawback of any single indicator in analyzing individual nodes. Section 3 proposes the methods *EIMI* and *RUMI* to evaluate the importance of nodes and identify the node role based on the selected indicators. Section 4 carries out experiments with 18 real networks and 3 kinds of model networks. Finally, Section 5 summarizes the paper.

## Analysis of Indicators' Correlations

Previous researches have only analyzed the correlation of a few indicators [16], such as the correlation between ego-betweenness centrality and betweenness centrality [17], [18]. The paper carries on a more thorough investigation into the correlations of 10 typical indicators listed in Table 1, where the indicators density and clustering coefficient are treated as one indicator since their formulas are the same.

**Table 1:** Overview of typical indicators.

Indicator		Equation	Indicator	Equation
(relative) degree		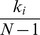	(ego) eigenvector centrality	The *i*th component of the eigenvector *x*of equation 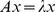
density/clustering coefficient		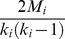	information centrality	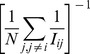
absolute (ego) betweenness centrality		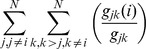	relative closeness centrality	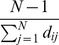
structural hole indicator	efficiency	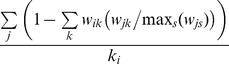	constrain	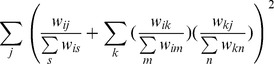
	effective size	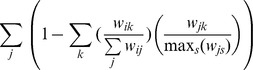	hierarchy	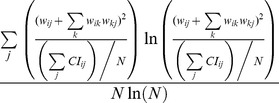

In this paper, an undirected unweighted network *G* is denoted *G*(*V*, *E*), where *V* is the set of nodes *v_i_* and *E* is the set of edges *e*(*v_i_*, *v_j_*). The number of nodes and edges are denoted *N* and *M*, respectively. *G*(*V*, *E*) can also be denoted an adjacency matrix *A* = (*a_ij_*)*_N_*_×*N*_, where *a_ij_* is equal to 1, if *e*(*v_i_*, *v_j_*) exists, otherwise *a_ij_* is equal to 0. The degree of node *v_i_* is denoted *k_i_*; the length of the shortest path between *v_i_* and *v_j_* is denoted *d_ij_*, where the number of the shortest paths that pass through node *v_k_* is denoted *g_ij_*(*k*). The information matrix of *G*(*V, E*) is denoted 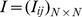, where 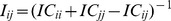, 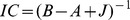, *B* is the diagonal matrix of node degree on the cater-corner, *J* is the identity matrix and the intensity matrix *W* is thus 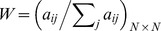.

In general, these indicators evaluate three different topology features of a node. First, the bonding feature, the most typical indicator is degree. Another indicator may be closeness centrality that evaluates the closeness of a node to the topological center of a network. Thus the node with the greatest closeness centrality could be considered as the most important core in a symmetry network like a star network. However, this ideal case is not satisfied by most real networks. Second, the bridge feature, including the indicators information centrality, betweenness centrality and four structural hole indicators. The first two indicators show the bridge performance of a node in the paths of a network. And the structural hole indicators that are efficiency, constrain, effective size and hierarchy are usually used together for comprehensive analyzing whether the neighbors of a node are well connected with each other. If the neighbors are not, there are structural holes around the node and the node must play the bridge role. Third, the topology feature of the sub-network around a node. Eigenvector centrality evaluates the overall importance of a node in the sub-network or the overall network, and density/clustering coefficient shows the density of edges of a node and its neighbors.

It is noteworthy that some indicators can be computed using the ego network of a node instead of using the topology of the overall network. In this case, the name of the indicator is usually added the prefix “ego”. The ego network of node *v_i_* is composed of *v_i_* (named ego), the nodes that *v_i_* connects with and the edges among these nodes. The two-layer ego network of *v_i_* is formed by the ego networks of the neighbors of *v_i_*. For instance, ego eigenvector centrality can be computed based on the two-layer ego network, while degree, ego betweenness centrality, ego information centrality etc. can be computed based on the one-layer ego network. And the sociological meanings of ego network have been widely studied in SNA [1], [7], [8].

**Figure 1:**
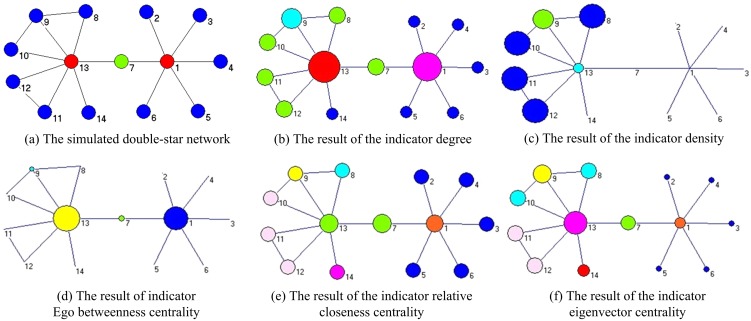
The performance of single indicator in evaluating the importance and the role of nodes of a double-star network.

First, the section assesses the performance of a single indicator in analyzing an individual node. Figure 1.(*a*) takes a simple double-star network as an example. Obviously, *v*_1_ and *v*_13_ are the core nodes that have the same degree but the different ego networks, and *v*_7_ is the bridge node in the network. In this case, any single indicator fails to identify the roles of these nodes and fails to distinguish their importance differences, as Figure 1.(*b*) - Figure 1.(*f*) show, where the size of a node represents its value of the corresponding indicator.

Then, the section analyzes the *Pearson* correlation and *Spearman* correlation between the 13 indicators including 3 ego indicators, based on 18 real networks [19]–[24], [31]–[36], [42]–[45], [48] and 3 kinds of model networks, including *ER*, *BA* and *WS*. And 10 different networks are randomly generated by the open source tool *Gephi* for each kind of model networks. The result is showed in Table 2 and Table 3. Because the correlation matrix of these indicators is symmetry, the bottom-left half of Table 2 and Table 3 shows the average *Spearman* and *Pearson* correlation coefficients between the indicators of these networks and the top-right half shows the corresponding standard deviation of the correlation coefficients of these networks. Table 2 and Table 3 also show the average correlation coefficients between degree and the other indicators of the model networks. And the corresponding standard deviations are similar to those of real networks. In this way, we could judge whether the correlation between two indicators are strong and stable in the different social networks.

**Table 2:** The Pearson correlation coefficients of 10 indicators and 3 ego indicators.

	degree	info. cen.	ego info. cen.	effect. size	bet. cen.	ego bet. cen.	eigen. cen.	ego eigen.	close. cen.	efficiency	density	hierarchy	constrain
**degree**	—	±0.10	±0.34	±0.15	±0.20	±0.15	±0.11	±0.18	±0.69	±0.38	±0.38	±0.37	±0.13
**info. cen.**	0.88	—	±0.39	±0.12	±0.17	±0.17	±0.16	±0.20	±0.71	±0.39	±0.42	±0.44	±0.14
**ego info. cen.**	0.55	0.47	—	±0.41	±0.35	±0.32	±0.32	±0.44	±0.53	±0.45	±0.50	±0.38	±0.52
**effect. size**	0.94	0.81	0.39	—	±0.16	±0.13	±0.20	±0.25	±0.68	±0.40	±0.34	±0.38	±0.13
**bet. cen.**	0.77	0.61	0.30	0.83	—	±0.22	±0.22	±0.25	±0.61	±0.34	±0.28	±0.30	±0.19
**ego bet. cen.**	0.83	0.66	0.35	0.89	0.82	—	±0.19	±0.23	±0.53	±0.34	±0.29	±0.28	±0.20
**eigen. cen.**	0.87	0.80	0.63	0.78	0.61	0.68	—	±0.33	±0.72	±0.36	±0.39	±0.32	±0.18
**ego eigen. cen.**	0.78	0.66	0.49	0.69	0.54	0.56	0.62	—	±0.60	±0.38	±0.39	±0.35	±0.21
**closeness cen.**	0.45	0.50	0.25	0.45	0.45	0.45	0.48	0.20	—	±0.40	±0.42	±0.43	±0.60
**efficiency**	−0.03	−0.11	−0.23	0.20	0.21	0.17	−0.11	−0.03	0.01	—	±0.41	±0.27	±0.32
**density**	−0.07	−0.04	0.20	−0.26	−0.23	−0.21	0.00	−0.03	−0.13	−0.79	—	±0.43	±0.31
**hierarchy**	−0.21	−0.35	0.10	−0.14	−0.07	−0.06	−0.18	−0.22	−0.06	0.30	−0.20	—	±0.47
**constrain**	−0.68	−0.76	−0.11	−0.72	−0.53	−0.51	−0.54	−0.56	−0.34	−0.28	0.40	0.35	—
**degree (BA)**	—	0.94	0.39	1.00	0.93	0.93	0.92	0.57	0.66	0.31	0.07	−0.34	−0.56
**degree (ER)**	—	0.96	0.30	1.00	0.92	0.95	0.91	0.69	0.74	0.11	0.03	−0.26	−0.68
**degree (WS)**	—	0.99	0.35	0.99	0.93	0.98	0.88	0.43	0.78	−0.02	−0.04	0.10	−0.75

**Table 3:** The Spearman correlation coefficients of 10 indicators and 3 ego indicators.

	degree	info. cen.	ego info. cen.	effect. size	bet. cen.	ego bet. cen.	eigen. cen.	ego eigen.	close. cen.	efficiency	density	hierarchy	constrain
**degree**	—	±0.14	±0.43	±0.13	±0.16	±0.17	±0.10	±0.19	±0.70	±0.46	±0.47	±0.37	±0.15
**info. cen.**	0.94	—	±0.43	±0.15	±0.19	±0.14	±0.16	±0.25	±0.74	±0.46	±0.48	±0.37	±0.14
**ego info. cen.**	0.45	0.43	—	±0.49	±0.46	±0.46	±0.38	±0.44	±0.54	±0.47	±0.51	±0.39	±0.52
**effect. size**	0.92	0.88	0.28	—	±0.11	±0.16	±0.20	±0.21	±0.68	±0.54	±0.50	±0.38	±0.12
**bet. cen.**	0.82	0.79	0.23	0.89	—	±0.13	±0.19	±0.21	±0.68	±0.50	±0.45	±0.39	±0.19
**ego bet. cen.**	0.87	0.87	0.26	0.94	0.90	—	±0.19	±0.20	±0.67	±0.51	±0.48	±0.37	±0.19
**eigen. cen.**	0.85	0.87	0.49	0.74	0.66	0.71	—	±0.38	±0.74	±0.42	±0.45	±0.31	±0.21
**ego eigen. cen.**	0.74	0.65	0.40	0.66	0.58	0.62	0.55	—	±0.56	±0.38	±0.39	±0.31	±0.22
**closeness cen.**	0.37	0.43	0.22	0.38	0.39	0.38	0.41	0.13	—	±0.49	±0.48	±0.40	±0.62
**efficiency**	−0.16	−0.14	−0.32	0.07	0.09	0.07	−0.21	−0.09	−0.06	—	±0.20	±0.39	±0.39
**density**	0.06	0.04	0.32	−0.18	−0.20	−0.20	0.15	0.02	−0.04	−0.91	—	±0.43	±0.37
**hierarchy**	0.00	0.00	0.28	0.04	0.06	0.07	0.01	−0.02	0.11	0.14	−0.16	—	±0.39
**constrain**	−0.80	−0.80	−0.15	−0.87	−0.80	−0.85	−0.63	−0.61	−0.31	−0.18	0.33	0.03	—
**degree (BA)**	—	0.99	0.39	1.00	0.96	1.00	0.92	0.56	0.90	−0.13	0.44	−0.32	−0.66
**degree (ER)**	—	0.99	0.3	1.00	0.95	1.00	0.91	0.75	0.88	−0.16	0.28	−0.16	−0.86
**degree (WS)**	—	0.94	0.35	0.99	0.92	0.99	0.85	0.56	0.77	−0.10	0.10	0.11	−0.90

The result shows that: (1) the indicators, including degree, (ego) information centrality, effective size, (ego) eigenvector centrality, absolutely (ego) betweenness centrality and relatively closeness centrality have positive correlations with each other. However, the correlations between some indicators are not very strong or stable, for instance the correlations between closeness centrality with the other indicators and the correlations between ego information centrality with the other indicators. (2) The correlation between the indicators of efficiency, density/clustering coefficient, hierarchy and constrain is not clear. (3) Two sets of the indicators referred in (1) and (2) have weak or negative correlations. These conclusions are satisfied by both the *Pearson* correlation and the *Spearman* correlation, thus form a sound foundation for selecting the multiple indicators with strong positive correlations.

## Node Analysis Based on Multiple Indicators

### Selection of multiple indicators

This paper proposes to select multiple indicators that have strong positive correlations to discover outstanding nodes. However, there are many different pairs of indicators that are strongly correlated with each other, for instance 12 pairs have correlation coefficients larger than 0.7 in Table 2. Therefore, in addition to the correlation between indicators, the paper also takes the following rules into consideration to select appropriate indicators: first, an indicator is preferred for a wider range of applications, if it has been widely applied and been proved to be useful for various social networks; second, selected indicators should evaluate different topology features of a node, and only one of the indicators evaluating a similar feature would be selected; third, an indicator that needs only the local topology of a node is preferred.

These selection rules are concluded as the correlation rule, the range-of-application rule, the diversity and concise rule and the local topology rule, respectively. Obviously, after the first indicator is selected, it would be easier to decide other candidates. The selection process is stated as follows.

Degree is the first choice, because degree is the most widely used indicator and has been proved to be essential for identifying the important nodes [47]. The node with great degree is more likely to be a core. Table 2 and Table 3 also show that the number of the indicators that strongly correlated with degree is the most. In descending order by the correlation coefficient, the indicators that have the correlation coefficients with degree larger than 0.70 in Table 2 and Table 3 are information centrality, effective size, (ego) betweenness centrality, and (ego) eigenvector centrality, while the correlation coefficients between closeness centrality and ego information centrality with degree are not very strong or stable. Thus, the other candidates should be selected from these strongly correlated indicators. Closeness centrality is not taken into consideration, because it may fail for asymmetry networks and it evaluates the similar feature with degree.

Second, ego betweenness centrality is selected as the indicator to evaluate the bridge function of a node. It has been proved in many applications that nodes with great (ego) betweenness are critical to information exchange and collaboration between two non-adjacent nodes. Ego-betweenness centrality is preferred for needing no global topology and its correlation coefficient with degree is also a little bigger than that between betweenness centrality and degree. The other candidates evaluating the bridge feature include (ego) information centrality and the structural hole indicators. However, although the correlation between information centrality and degree are very high, the correlation between the ego version of information centrality and degree is low and unstable, as Table 2 and Table 3 show. As for the four structural hole indicators, only effective size is strongly correlated with degree. We do not select effective size, because it is usually used along with other structural hole indicators and has not been widely applied, especially for analyzing the newly emerged large social networks.

Third, (ego) eigenvector centrality is selected as the indicator to characterize the “global” prominence of a node in the overall network or a sub-network. The indicator shows how well connected a node is to other important nodes, therefore great (ego) eigenvector centrality would enforce the status of a node as a core or a bridge. For instance, a bridge is more important, if it connects with other important nodes. Because Table 2 and Table 3 show that eigenvector centrality has stronger correlation with degree than ego eigenvector centrality does, eigenvector centrality could be better according to the correlation rule. But ego eigenvector centrality is preferred due to the local topology rule, and once it is selected, all of the three indicators can be computed without global topology. This property will greatly improve the adaptability and the efficiency of our method. Thus, it is a difficult choice between eigenvector centrality and ego eigenvector centrality and their performance will be further compared in the experiments. The other candidate density/clustering coefficient is omitted because it is not positive correlated with degree.

Finally, this paper selects degree, ego betweenness centrality and (ego) eigenvector centrality as the multiple indicators to assess different characteristics of a node, including whether a node has many ties with other nodes, whether it has more control over the interactions between other nodes, whether it connects with other important nodes well. To be more clearly, we summarize the selection process in Table 4 that generally states the topology feature of an indicator, whether the indicator is selected and the reason based on the selection rules.

**Table 4:** The overview of the indicator selection process.

Topology Feature	indicator	Selected	Reason
Bonding feature	degree	**Yes**	The range-of-application rule
	closeness centrality	**No**	The correlation rule and the concise rule
Bridge feature	(ego) betweenness centrality	**Yes**	The correlation rule, the diversity rule and the local topology rule
	(ego) information centrality	**No**	The local topology rule, the correlation rule and the concise rule
	Four structural hole indicator	**No**	The correlation rule and the range-of-application rule
Sub-network feature	(ego) eigenvector centrality	**Yes**	The correlation rule, the diversity rule and the local topology rule
	Density/clustering coefficient	**No**	The correlation rule and the concise rule

In general, these indicators would be highly adaptable to various networks. The selection rules and the process is a guideline, which can be revised for a specific application by selecting indicators that are very effective for the application, and can also be extended by adding new indicators as candidates.

The complexity for computing these indicators is analyzed as follows. The computation complexity of degree is *O*(*M*); the computation complexity of betweenness centrality is *O*(*N* ^3^) using the *Floyd* algorithm [25]; and the computation complexity of eigenvector centrality is also *O*(*N* ^3^) using the *QR* algorithm [26] and the inverse power iteration method [27]. If all of the selected indicators are computed based on the ego-network, the complexity can be greatly reduced. When using ego-networks, the average size of each node's one-layer ego-network is (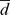 *+*1) and the size of its two-layer ego-network is ((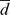
+1)^2^ *+*1), where 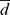 is the average degree of all of the nodes. Therefore, the computation complexity of ego-betweenness centrality is reduced to *O*(*N*×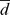 ^3^) and the complexity of eigenvector centrality is reduced to *O*(*N*×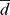 ^2×3^) based on two-layer ego-networks, while 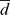 and 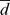 ^2^ are far smaller than *N* in most cases.

### Evaluation of node importance

This paper decides a node's importance as the normalized value sum of the three indicators, and the method is termed as *EIMI* (*Evaluation of Importance based on Multi-Indicator*) method. The normalized value of the degree, the ego-betweenness centrality and the eigenvector centrality of *v_i_* is denoted 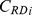, 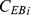
and 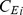 respectively. Then, the importance 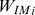 of *v_i_* is thus:
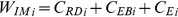
(1)


Obviously, the performance of *EIMI* can be further improved by appointing the different weights *α*, *β* and *γ* for the three indicators, respectively. However, automatic optimization of weights is still an open problem in machine learning, and the automatic process depends on the prior knowledge that is quite rare for social networks, while manually appointing appropriate weights relies on an expert's experience that is expensive to obtain and varies with the networks. Taking these factors into consideration, we simplify the method by setting the weights of the three indicators as equal.

Compared with other methods, such as *PageRank*, *EIMI* not only shows the importance of nodes but also shows how the importance is constituted by showing the values of these different indicators. This feature will be helpful to understand the reason why a node is important when we refer to the sociological means of these indicators.

### Identification of node role

Studies of social network have shown that the importance of a node alone isn't enough to identify its role [28].

Thus, this paper proposes the *RUMI* (*Role jUdgment based on Multi-Indicator*) algorithm that selects the core candidates from the nodes with great degree and the bridge candidates from the nodes with great ego-betweenness centrality. The role of a candidate node is finally determined according to the difference between its indicators relationship with the statistical correlation of the overall network. If the node shows the more significant characteristics of connecting non-adjacent nodes together and of connecting with other important nodes, it is recognized as a core or a bridge.

*RUMI* firstly sorts all of the nodes in descending order by degree, ego-betweenness centrality and (ego) eigenvector centrality separately. The rank of a node *v_i_* is denoted 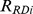, 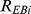 and 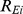 respectively. And the nodes with the same value of an indicator have the same rank.

Because the selected three indicators have strong correlations with each other, 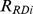, 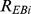 and 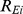 of an ordinary node *v_i_* should be very similar. Thus, the rank differences 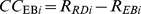 and 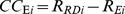 of the node *v_i_* should be very small. Therefore, the general correlation of the overall network can be evaluated by the average rank difference of all of the nodes. The average rank difference of the network is computed as follows:
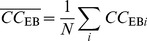
(2)

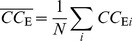
(3)


If 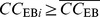, its means that the rank of ego-betweenness centrality of *v_i_* is higher than the rank of degree; thus, the node not only connects with many other nodes, but also shows a more significant feature of information exchange. If 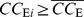, its means that the rank of eigenvector centrality of *v_i_* is higher than the rank of degree; thus, the node shows a more significant feature of connecting with other important nodes.

Finally, the role of a selected node *v_i_* is determined according to the following rules:

1. Iff 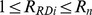, 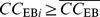 and 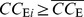, *v_i_* is a core node.
2. Iff 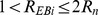, 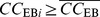, 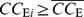 and *v_i_* is not a core, *v_i_* is a bridge node.

where the threshold *R_n_* is adopted to decide the number of candidate nodes. If the average degree 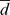 of a network is too small or the network is a scale-free network, we propose to set the value of *R_n_* as the inflection value of the degree distribution curve of the network. Otherwise, *R_n_* is preferred to be 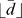. Because some cores also have very high rank of ego-betweenness centrality, the number of bridge candidates is set larger than that of core.

Therefore, the role identification process of *RUMI* is: first, compute the values of multiple indicators of each node; second, rank the node according to their indicators separately, see the step 10 and step 11 in Table 5; third, compute the rank difference of each node and the average difference of the network, see the step 12 to step 20 in Table 5; finally, select the candidates of core and bridge, then identify the role of selected nodes according to the identification rules, see the step 21 to step 35 in Table 5. The pseudocode of *RUMI* is listed in Table 5. *RUMI* just selects 3*R_n_* nodes for role determination and its computation complexity is *O*(*N*×*logN*) [30] and the complexity for computing indicators is *O*(*N* ^3^) that could be reduced to *O*(*N*×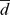 ^2×3^) if the computation of the indicators only depends on ego-networks.

**Table 5:** The pseudocode of *RUMI.*

Pseudocode	Description
1. **Input**: network *G*(*V*,*E*);	
2. **Output**: arrays core_nodes[], bridge_nodes[];	*Arrays to record the detected cores and bridges*
3. **Begin**	
4. Set *N* = |*V*|, *M* = |*E*|;	*Number of the nodes and the edges of the network G*
5. Set *integer * 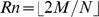 ;	*The number of the candidate cores and bridges*
6. Set *double* avg_egobet_dif = 0, avg_eigen_dif = 0;	*The average rank difference * 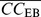 * and* 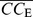 *of the network G*
7. Set *integer* core_num = 0, bridge_num = 0;	*Number of detected cores and bridges at current stage of RUMI*
8. Set *array* node_indicator[*i*] [3], node_rank[*i*] [3];	*The value and the rank of three indicators of all nodes*
9. Set *array* temp[*N*];	*Temporary array used in the computing process*
10. node_indicator = *Calculate_Indicator*(*G*(*V*,*E*));	*Computing the indicators*' *value of all of the nodes*
11. node_rank = *Get_Rank*(node_indicator);	*Ranking all of the nodes based on the corresponding indicator*
12. **for** each node *i* ∈ *V* **do**	
13. temp[*i*] = node_rank [*i*] [2];	*Recording the rank of ego-betweenness of node v_i_*
14. node_rank[*i*] [2] = node_rank[*i*] [1]-node_rank[*i*] [2];	*Computing the rank difference of degree and ego-betweenness of v_i_*
15. node_rank [*i*] [3] = node_rank [*i*] [1] - node_rank[*i*] [3];	*Computing the rank difference of degree and eigenvector of v_i_*
16. avg_egobet_dif + = node_rank [*i*] [2];	*Summing up the rank difference CC_EB_ of all nodes*
17. avg_eigen_dif + = node_rank [*i*] [3];	*Summing up the rank difference CC_E_ of all nodes*
18. **end**	
19. avg_egobet_dif = avg_egobet_dif/*N*;	*Computing the average rank difference* 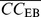 *of G*
20. avg_eigen_dif = avg_eigen_dif/*N*;	*Computing the average rank difference * 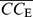 * of G*
21. **for** each node *i* ∈ *V* **do**	
22. **if** node_rank[*i*] [1]> = 1 **and** node_rank[*i*] [1]< = *Rn*	*Selecting nodes with great degree as core candidates*
23. **and** node_rank [*i*] [2] > = avg_egobet_dif	*Detecting cores from the candidates based on the rank differences*
24. **and** node_rank [*i*] [3] > = avg_eigen_dif	
25. core_nodes[core_num] = *i*;	*Recording the detected cores*
26. core_num ++;	
27. **end**	
28. **if** temp[*i*]> = 1 **and** temp[*i*]< = 2*Rn*	*Select nodes with great ego-betweenness as bridge candidates*
29. **and** node_rank [*i*] [2] > = avg_egobet_dif	*Detecting bridges from the candidates based on the rank differences*
30. **and** node_rank [*i*] [3] > = avg_eigen_dif	
31. **and** *i* !∈ core_nodes	*The bridge cannot be a core simultaneously*
32. bridge_nodes[bridge_num] = *i;*	*Recording the detected bridges*
33. bridge_num ++;	
34. **end**	
35. **end**	
36. **return** core_nodes, bridge_nodes;	
37. **End**	

**Figure 2:**
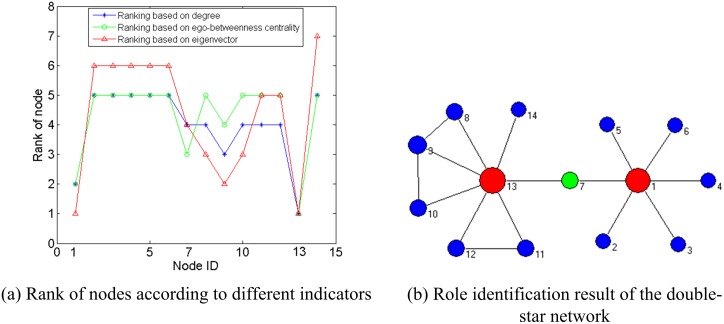
The process of role identification of *RUMI* of the *double-star* network, where a red node represents a core node and a green node represents a bridge node.

Figure 2 shows the process of role identification of the *double-star* network. Figure 2.(*a*) is the rank of each node according to each indicator. It can be seen that *v*_13_ and *v*_1_ always have the highest ranking or the second-highest ranking, thus they are undoubted cores; *v*_7_ shows its significance for connecting other node-pairs, because its rank of ego-betweenness centrality is much higher than that of degree. Figure 2.(*b*) shows the result of role identification, where the red ones represent the detected cores, the green represent the detected bridge, and the size of a node represents its importance computed by *EIMI*. Compared with Figure 1, *RUMI* algorithm correctly identifies the roles of the nodes *v*_1_, *v*_7_ and *v*_13_.

## Experiments and Analyses

The paper adopts various kinds of social networks in the experiments, including: *Karate* [19], spare time relationship of members of a Karate club; *Football* [21], game relationship of US college football teams in the regular season; *Dolphins* [22], frequent associations between bottlenose dolphins in a group; *Lesmis* [24], coappearances of characters in the novel “*Les Miserables*”; *Adjnoun* [20], juxtapositions of words in the novel *David Copperfied*; *Polbooks* [23], network of books about US politics sold by the Amazon.com; *Dining_table_partners* [31], dining-table partnership in a dormitory at a Training School; *Freemans*_1 [32], the relationships of the early researchers of SNA; *literature*_1976 [34], the critical attentions among literary authors and critics; *Sawmill* [35], communication network between employees of a sawmill; *Grassland* [33], *Seagrass* [36] and *Ythan* [33], the predatory interactions among species in a place of U.K., of winter's seagrass and of Ythan Estuary parasites respectively; series *World_trade* networks, the trade relationship of four kinds of goods [48], [29] between nations; *P2p*-1 [42], a sequence of snapshots of the Gnutella peer-to-peer file sharing network; *UCIonline* [43], the online message network of the students of UC. Irvine; *USpowerGrid* [44], the power grid in USA; *Zewail* [45], the reference relationship between papers. And the networks of each synthetic *ER, BA and WS* model are randomly generated ten times by the open source tool *Gephi*'s *BA* Scale free Model B, *Gephi*'s *ER G*(*n, p*) Model and by *Gephi*'s WS small world Model Alpha, respectively. Detail information of these networks is listed in the supplementary Table S1. Because the proposed method aims at analyzing an undirected no-weighted social network, the direction and weight of edges of some networks is not adopted in the following experiments.

These networks contain different types of social actors including: different kinds of individuals, such as the *karate*, *dolphins* and *USpowergrid* network; or an organization, such as the *football* network; or a nation, such as the *world_trade* networks. Because the available social networks have no sufficient prior knowledge about the importance and the role of nodes, we evaluate the results mainly by visualizing the overall network for detail observation. Because of the difficulties in visualizing a large network, the adopted networks are mainly of small size. And 4 large networks are adopted to further verify the performance of the proposed methods, where the 1395 nodes with zero degree of *Zewail* network are eliminated. Finally, 18 real social networks and 3 kinds of model networks are adopted in the experiments. These networks are the same with those of Section 2.

Table 6 is the overview of these networks, where the *world_trade of manufactures of metal* is stated as the representation of other trade networks. According to the degree distribution, the *karate*, *adjnoun*, *literature_1*976, *Sawmill*, *grassland*, *lesmis*, *ythan*, *polbooks*, *Zewail*, *UCIonline* and *USpowerGrid* networks are similar with a scale-free network; the *football* and *Freeman*-1 networks are nearly a full-interconnection network that is similar with a small-world network; the network *dining_table_partner* and *world_trade* are similar with a *ER* network; the other networks, including *dolphins, seagrass* and *p*2*p-*1 show no clear degree distribution pattern, thus could not be classified.

**Table 6:** The overview of networks and the results of role identification.

Networks		*N*	_ 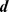	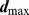	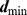	Num. of cores	Num. of bridges
Real networks	*karate* [19]	34	4.59	17	1	4/4(4)	5/4(4)
	*Football* [21]	115	10.66	12	7	37/44(37)	0/0(0)
	*dolphins* [22]	62	5.12	12	1	11/12(11)	1/0(0)
	*lesmis* [24]	77	6.59	36	1	7/7(7)	4/4(4)
	*adjnoun* [20]	112	7.59	49	1	12/12(12)	6/6(6)
	*polbooks* [23]	105	8.4	25	2	16/16(16)	1/1(1)
	*Dining_table_partners* [31]	26	3.19	7	1	5/5(5)	2/3(2)
	*Freemans_*1 [32]	34	24.41	33	9	11/11(11)	0/0(0)
	*literature_1*976 [34]	35	4.57	12	1	7/5(5)	1/1(1)
	*Sawmill* [35]	36	3.44	13	1	3/2(2)	4/1(1)
	*grassland* [33]	88	3.11	17	1	4/4(4)	2/1(1)
	*seagrass* [36]	49	9.10	17	2	17/17(17)	0/0(0)
	*ythan* [33]	135	8.83	54	1	10/10(10)	6/6(5)
	*World_trade of metal* [48]	80	21.875	77	4	27/27(27)	6/6(6)
	*p*2*p-*1 [42]	10876	7.355	103	1	8/8(8)	6/6(6)
	*UCIonline* [43]	1899	14.574	255	1	15/15(15)	13/13(13)
	*USpowerGrid* [44]	4941	2.669	19	1	2/2(2)	2/2(2)
	*Zewail* [45]	6752	1.697	23	1	4/4(4)	7/7(7)
Model networks	*WS* [2]	200	4	8	3	46/45(40)	0/0(0)
	*ER* [15]	200	4	10	1	39/39(37)	0/0(0)
	*BA* [3]	200	4	18	2	8/7(7)	11/4(4)

### Evaluation of node importance based on multiple indicators

First, the performance of the *EIMI* method based on global topology (termed global*-EIMI*) and the *EIMI* based on ego network (termed ego*-EIMI*) is analyzed.

In these networks, the series *world_trade* networks have the most complete prior knowledge of node importance that equals the sum of a nation's trade value of the good with other countries. We have also tried to generate a synthetic hierarchy network with complete prior knowledge. In the hierarchy structure, the importance of a node is highly related with its hierarchy, but the topology of around non-leaf nodes is similar. To tackle this problem, we assume that a high hierarchy node has possibility to connect with low hierarchy nodes. However, the attempt is not successful. One major reason may be that the additional connections obscure the importance differences of nodes. For instance, it is arguable to tell the differences between a low hierarchy node with a high hierarchy node, where the former connects with some much higher hierarchy nodes and the latter has no additional connections instead of those with its immediate father and subordinates.

Therefore, we evaluate the performance of global*-EIMI* and ego*-EIMI* based on the series *world_trade* networks of different goods, and the *glass*, *tobacco* and *grain* networks have 212, 199 and 214 nodes respectively, with 3837, 2596 and 3993 edges respectively. Similar with the previous ranking studies [49], [50], Table 7 concentrates on the consistency of the Top 10 results of ego-*EIMI* with the prior knowledge. 80%, 70%, 80% and 60% of its TOP 10 nations of the four different *world_trade* networks are consistent with the corresponding prior knowledge. The performance of global-*EIMI* is similar to that of ego-*EIMI*. It can be seen that the performance of *EIMI* is acceptable.

**Table 7:** Comparison the Top 10 results of ego-*EIMI* with the prior knowledge of trade value of different goods, where the country name with the blue and italics font of ego-*EIMI* appears in the TOP 10 of trade value too.

Rank	manufactures of metal		grain		glass		tobacco	
	ego-*EIMI*	Trade value	ego-*EIMI*	Trade value	ego-*EIMI*	Trade value	ego-*EIMI*	Trade value
1	***Germany***	USA	***USA***	USA	***China***	China	***Germany***	Germany
2	***Italy***	Germany	***Thailand***	France	***USA***	Germany	***USA***	Holland
3	***USA***	Japan	***India***	Japan	***France***	USA	***Spain***	USA
4	***U. K.***	U. K.	***France***	Canada	***Germany***	France	***France***	Italy
5	***China***	Italy	***Canada***	Thailand	***U. K.***	Japan	***Belize***	Japan
6	***Japan***	Canada	China	Germany	***Italy***	Italy	Switzerland	Belgium
7	***France Mon.***	China	Pakistan	Belize	Turkey	Belgium	***Belgium***	France
8	***Netherlands***	Netherlands	***Germany***	Mexico	Spain	Hong Kong	Denmark	Belize
9	Belgium	France Mon.	Italy	Ukraine	***Japan***	U. K.	U. K.	Poland
10	Spain	Mexico	***Ukraine***	Egypt	***Belgium***	Korea	Greece	Spain

**Figure 3:**
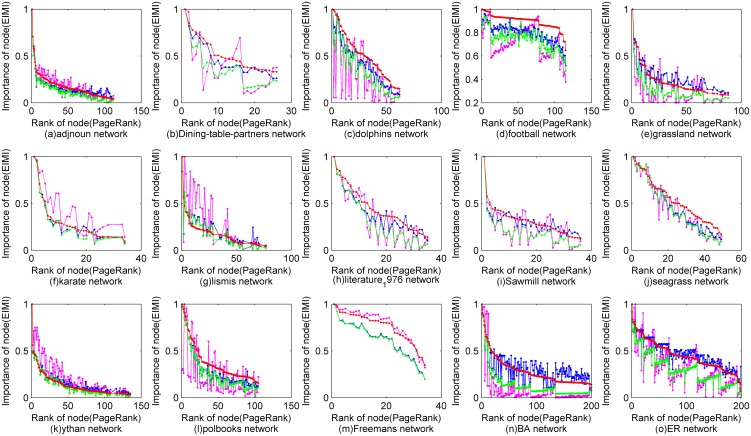
Comparison of the ranking result of *EIMI* with that of *PageRank* and *HITS* for 15 small networks. The importance of a node computed by global*-EIMI*, ego*-EIMI*, *HITS* and *PageRank* is separately colored with green, blue, purple and red.

**Figure 4:**
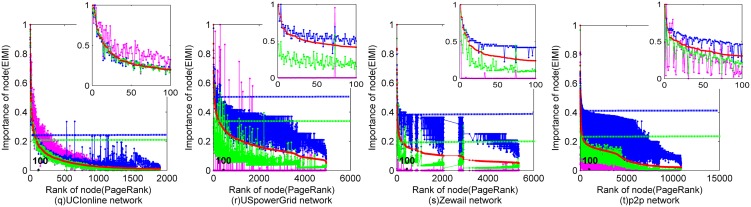
Comparison of the ranking result of *EIMI* with that of *PageRank* and *HITS* for 4 large networks. The importance of a node computed by global*-EIMI*, ego*-EIMI*, *HITS* and *PageRank* is separately colored with green, blue, purple and red. The top-right small figure compares the importance of the TOP 100 nodes of *PageRank* with that of other methods. the straight lines in the bottom-left big figure shows the lowest importance value of these TOP 100 nodes, which is computed by global*-EIMI*, ego*-EIMI* respectively.

For the other networks that have no or incomplete knowledge about node's importance, recent studies usually take the widely-used method *PageRank* for comparison [40], [41]. The paper follows this way and takes another typical method *HITS* [46] for further comparison. In Figure 3 and Figure 4, *x*-axis shows the rank of nodes of *PageRank*; *y*-axis shows the importance of a node computed by global*-EIMI*, ego*-EIMI*, *HITS* and *PageRank*, which are separately colored with green, blue, purple and red. Therefore, the consistency of the curve trends reflects the similarity of the ranking results of the different methods. Because there are a large number of nodes visualized in Figure 4, the importance differences of a small part of the nodes may shelter other details of the figure. Because high ranking nodes are more important in practice, Figure 4 shows a top-right small figure that compares the importance of the TOP 100 nodes of *PageRank* with that of global*-EIMI*, ego*-EIMI* and *HITS*. Meanwhile, the bottom-left big figure shows the two lines corresponding to the lowest importance value of these TOP 100 nodes, which is computed by global*-EIMI*, ego*-EIMI* respectively. Therefore, if a node's importance is higher than the line, the node should be ranked higher by global*-EIMI* or ego*-EIMI*.

It can be seen that ego-*EIMI* performs quite similar with *PageRank* for most networks even without using the global topology that is required by *PageRank*. And the ranking results of *PageRank* and *EIMI* are quite different with that of *HITS*. It is because that both *EIMI* and *PageRank* tend to rank a node with the greater degree higher [47], while the betweenness centrality and the eigenvector centrality of the node is highly correlated with its degree. Therefore, ego-*EIMI* could be applied as a replacement of *PageRank*, especially when no global topology is available or the total computation time is limited.

**Figure 5:**
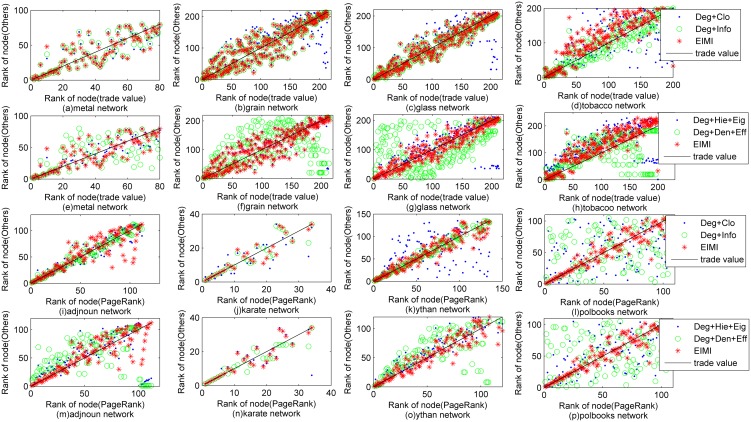
Performance of *EIMI* under different indicator selections of the series *world_trade* networks with trade value as the ground truth, and the *adjnoun*, *karate*, *ythan* and *polbooks* networks with the result of *PageRank* for comparison, where the shape and color of a node correspond to its importance under different indicator selections.

Second, we analyze the effectiveness of *EIMI* by comparing with the result of other multiple indicators. Figure 5 shows the ranking results of the series *world_trade* networks with trade value as the ground truth, and the *adjnoun*, *karate*, *ythan* and *polbooks* networks with the result of *PageRank* for comparison. Figure 5.(*a*)–(*d*) and Figure 5.(*i*) – (*l*) compares the selections of other centrality indicators, where the blue prismatic represents the result of the indicators of degree and closeness centrality, the green circle represents the result of degree and information centrality, and the red star represents the result of ego-*EIMI*. Figure 5.(*e*) – (*h*) and Figure 5.(*m*) – (*p*) compares the selections of some weak or negative correlated indicators, where the blue prismatic represents the result of degree, hierarchy and eigenvector centrality, the green circle represents the result of degree, density/clustering coefficient and efficiency, and the red star represents the result of ego-*EIMI*. The results of other networks are similar to Figure 5.

It can be seen that the indicator selection of *EIMI* performs quite well and stable. For instance, although the selection of degree and information centrality is a little better than *EIMI*, its performance for the *world_trade of tobacco* and *polbooks* network is unacceptable. And, the multiple indicators with weak or negative correlation would greatly reduce the performance.

Third, we show the difficulties in deciding appropriate weight *α*, *β* and *γ* for the three indicators. The most appropriate weights of *α*, *β* and *γ* computed by the Generalized Linear Regression method for the *world_trade* of *manufactures of metal*, are 0.14, 0.72 and −0.03, those of *glass* network are 0.30, 0.89 and −0.13, those of *tobacco* network are 0.42, 0.39 and −0.16, and those of *grain* network are −0.20, 0.72 and 0.16. Thus, it is still a hard job to find a robust method that decides each indicator's weight automatically, particularly in the case that no prior knowledge is available.

### Node role identification

Finally, we analyze the effectiveness of the *RUMI* method in determining the role of nodes. Table 6 shows the number of cores and bridges identified by *RUMI* based on global topology (termed global*-RUMI*) and based on ego network (termed ego*-RUMI*). For instance, the number of cores of the *karate* are “4/4(4)”, which means that global*-RUMI* identified 4 cores, so does ego*-RUMI*, and the results have 4 cores in common. It can be seen that the result of ego*-RUMI* is remarkably consistent with that of global*-RUMI*. Because the 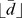 of the *Zewail* network is too small, its value of *R_n_* is set to be the inflection value of its degree distribution curve, which is 4. The value of *R_n_* of the other networks is equal to 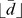.

**Figure 6:**
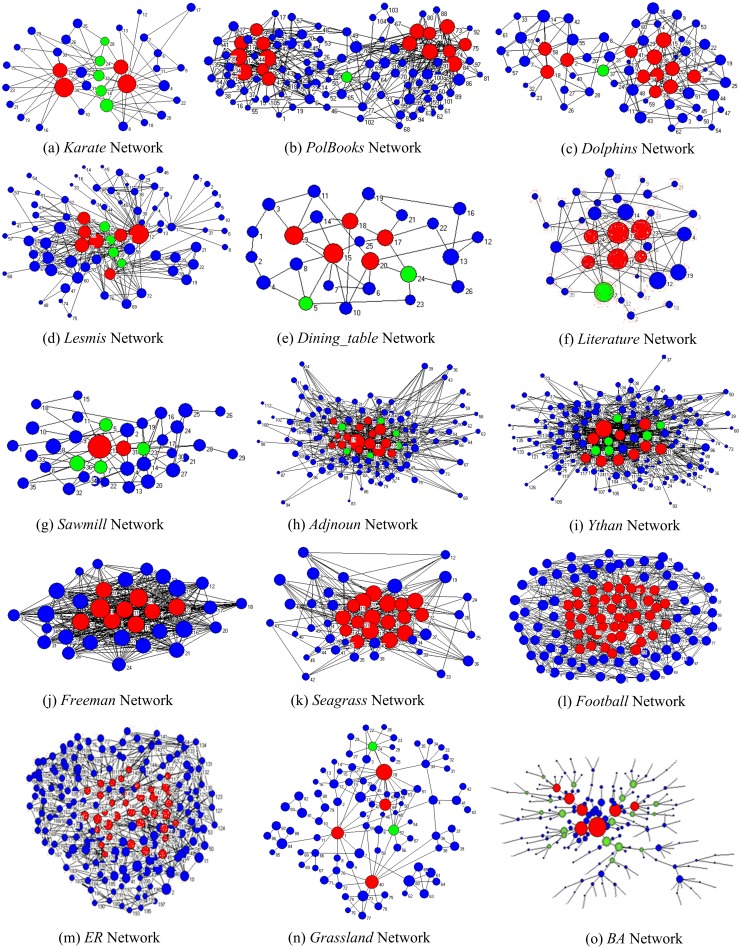
Role identification results of 15 networks of global-*RUMI*, where a red node represents a core node, a green node represents a bridge node and the size of a node corresponding to its importance decided by *EIMI*.

Figure 6 visualizes the role identification results of global-*RUMI* of 13 real networks and *BA*, *ER* model networks, where a red node represents a core node, a green node represents a bridge node and the size of a node corresponds to its importance decided by global-*EIMI*. The result of *WS* networks is similar with that of *ER* networks.

As we can see from Figure 6, *RUMI* has detected the core nodes and the bridge nodes quite well, especially for the BA type networks. Nearly all of the detected cores and all of the bridges take the significant position in the networks. Detail analysis is as follows: (1) for the *karate*, *polbooks*, *dolphin* and *lesmis* networks that have the clear two-community structure, the detected cores locate in the heart of each community and the different communities are connected through the bridge area that includes all of the detected bridges. (2) For the *adjnoun*, *Dining-table-partners*, *literature*-1976, *Sawmill* and *ythan* networks that have no obvious community structure, *RUMI* detects only one heart area that contains nearly all of the cores and the detected bridges connect the heart area with the outskirt area of the network. (3) For the *Freeman-*1, *seagrass*, *football*, WS and *ER* networks, *RUMI* detects the cores aggregating together and doesn't find any bridge. This result is complied with the feature of a highly connected network. (4) For the *grassland* network with multiple centers, *RUMI* detects the most significant centers and a bridge locating at the critical connecting position; for the *world_trade* networks, the detected cores aggregating together with a few bridges located around them; for the *BA* network with the tree structure, *RUMI* not only detects the node roles, but also discovers the hierarchy of cores or bridges if the importance of nodes decided by *EIMI* is taken into consideration.

Although it is very difficult to visualize a large network for detail observation, Table 6 shows that *RUMI* has successfully distinguished a few interesting nodes for further analysis. It is more satisfying that the results of ego-*RUMI* and global-*RUMI* are the same.

The experiments show that the proposed method is more suitable to analyze the BA type networks. As for the ER and the WS type networks, it identifies about 20% nodes as results, which may be too many. The reason lies in that according to the basic idea of the method, if a few nodes do not comply with the statistical relationship of the overall network and most of the nodes do, the proposed method would make more sense. A scale-free network has some very high degree nodes that unlikely exist in an ER or a WS network. Previous researches referred a scale-free type network as a degree *heterogeneous* network. Because of the strong correlations between degree with ego-betweenness centrality and eigenvector centrality, these topologies selected by the paper are also *heterogeneous* in this kind of networks.

We find other cases that our method is not so good. Some local cores or local bridges that locate at the sparse parts of a network are not detected, such as the *grassland* network, although most of the global ones are detected. In this case, we propose to regard a sparse part as a new network for further analyses, if the part is interesting and critical.

The performance of other combinations of multiple indicators with strong correlations are also evaluated, including degree+ego-betweenness centrality+clossness centrality, degree+ego-betweenness centrality+information centrality and degree+ego-betweenness centrality+effective size. However, these combinations could not identify node roles effectively. For instance, no core or bridge is detected by these multiple indicator combinations for the *Karate* network.

**Figure 7:**
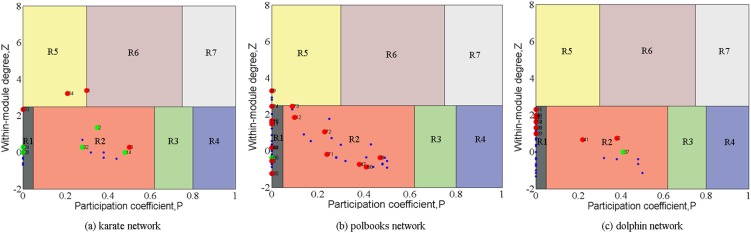
Comparison of Role identification results of *RUMI* with that of ref. [38]. According to the process of ref. [38], the *karate*, *polbooks* and *dolphin* networks that have clear community structure are selected.

We also compare our method with the functional cartography method, another role detection method proposed by ref. [38]. Figure 7 visualizes the detection result of the functional cartography. Because community detection is a prerequisite of the functional cartography, the performance of the functional cartography is highly sensitive to the community quality. Thus, we select three networks that have very clear community structure, including the *karate*, *polbooks* and *dolphin* networks. And we manually adjust the two communities of these networks discovered by *FN* [39] to ensure the high-performance of the functional cartography. According to the functional cartography, in general, a node locates at the R3 and R4 zone is a bridge, while a node locates at the R5, R6 and R7 zone is a core. In Figure 7, the color of a node corresponding to its role detected by *RUMI*. It can be seen that *RUMI* is better than the method of ref. [38]. A possible explanation may be that the functional cartography aims at analyzing metabolic networks whose topology feature is quite different from that of social networks.

Summarily, the experimental results show the good performance of the proposed method in evaluating the importance and the role of nodes, especially for the heterogeneous networks. Moreover, the result based on the global topology of a network is highly consistent with that based on the ego networks of nodes.

## Conclusions

On the basis of correlation analyses of typical indicators, the paper proposes the methods to evaluate the importance and the role of nodes based on multiple indicators with strong correlations. The experimental results show the good performance of the proposed methods in analyzing the heterogeneous networks. And the result based on the global topology is highly consistent with that based on ego networks. Therefore, the proposed method would be adaptable to the large, time-varying network whose precise global topology is always absent, such as the Internet and the social network of *FaceBook*.

The paper also shows that the performance of the role detection method may vary with fields. For instance, the sound functional cartography method is not good at analyzing some social networks. We guess that it is caused by the topology differences between the networks of different fields. But, what kinds of differences are there, why these differences exist and how the differences affect the role detection result, still need to be explored in the future work. Moreover, it is still an open problem to decide the appropriate weight of different indicators for role identification. The automatically method without any or just a little prior knowledge is preferred.

## Supporting Information

Table S1 **Detail information about the networks.**
(DOC)
