# Supplementary material for: Identifying Node Role in Social Network Based on Multiple Indicators
Source: PLoS One. 2014 Aug 4;9(8):e103733. doi: 10.1371/journal.pone.0103733 (PMC4121239; doi:10.1371/journal.pone.0103733)
Supplement: Table S1 — Detail information about the networks. (DOC) [file pone.0103733.s001.doc]

**Table S1 Detail information about the networks**

| **Networks** | | **Description** | **Download website** |
| --- | --- | --- | --- |
| Real networks | *karate*[19] | spare time relationship of members of a Karate club, where a node represents a member and an edge means two members spent their spare time together | http://networkdata.ics.uci.edu/data/karate/ |
| *Football*[21] | game relationship of US college football teams in the regular season fall 2000, where a node represents a team and an edge means two teams have game in the season | http://vlado.fmf.uni-lj.si/pub/networks/data/sport/football.htm |
| *dolphins*[22] | frequent associations between bottlenose dolphins living off Doubtful Sound, New Zealand, where a node represents a dolphin in a community and edges show frequent associations between dolphins | http://networkdata.ics.uci.edu/data/dolphins/ |
| *lesmis*[24] | coappearances of characters in the novel *Les Miserables*, where a node represents a character and the edges connect any pair of characters that appear in the same chapter | http://www-personal.umich.edu/~mejn/netdata/lesmis.zip |
| *adjnoun*[20] | juxtapositions of words in a corpus of the novel *David Copperfied*, where the nodes represent the most commonly occurring nouns and the most commonly occurring adjectives in the novel and an edge connects any two words that appear adjacent to one another at any point in the book | <http://networkdata.ics.uci.edu/data/adjnoun/> |
| *polbooks*[23] | network of books about US politics sold by the Amazon.com published around 2004 presidential, where a node represents a book and edges represent frequent copurchasing of books by the same buyers | http://www-personal.umich.edu/~mejn/netdata/polbooks.zip |
| *Dining_table_partners*[31] | dining-table partnership in a dormitory at a New York State Training School, where a node represents a person and an arc connects any two persons that one are willing to sit adjacent to the other | <http://vlado.fmf.uni-lj.si/pub/networks/data/esna/dining.htm> |
| *Freemans_*1[32] | the personal relationships among the early researchers of SNA | [http://toreopsahl.com/datasets/#FreemansEIES](http://toreopsahl.com/datasets/#FreemansEIES) |
| *literature_1*976[34] | the critical attention among a set of Dutch literary authors and critics in 1976, where an arc connects two people if the first has commented on the work of the second in an interview or review | <http://vlado.fmf.uni-lj.si/pub/networks/data/esna/literature.htm> |
| *Sawmill*[35] | communication network between all employees of a sawmill, where two employees were linked in the network if they rated another as high frequency | <http://vlado.fmf.uni-lj.si/pub/networks/data/esna/sawmill.htm> |
| *grassland*[33], *seagrass*[36], *ythan*[33] | the predatory interactions among species in a place of UK, of winter's seagrasses and of Ythan Estuary with parasites respectively, where each node represents a species and if species *i* preys on species *j*, then a directed link is drawn from *j* to *i* | <http://pil.phys.uniroma1.it/~gcalda/cosinsite/extra/data/foodwebs/ythan.txt>, seagrass.txt and grassland.txt |
| *World_trade* [48,29] | the international trade relationship between nations, where a node represents a nation and the weight of an edge shows the total trade value of the corresponding good between two countries, including, *manufactures of metal*, *grain*, *glass* and *tobacco* | http://vlado.fmf.uni-lj.si/pub/networks/data/esna/metalWT.htm  <http://comtrade.un.org/monthly/Bulk/Bulk.aspx> |
| *p*2*p-*1[42] | a sequence of snapshots of the Gnutella peer-to-peer file sharing network from August 2002, where a node represent a host and the edges represent connections between the Gnutella hosts | <http://snap.stanford.edu/data/p2p-Gnutella04.html> |
| *UCIonline*[43] | the online message network of the students of UC. Irvine from April to October 2004, where a directed tie is established from one student to another if one or more messages have been sent from the former to the latter and the weight of a tie is defined as the number of messages | <http://toreopsahl.com/2009/04/03/article-clustering-in-weighted-networks/> |
| *USpowerGrid*[44] | the high-voltage power grid in the Western States of USA, where the nodes are transformers, substations, and generators, and the ties are high-voltage transmission lines | <http://vlado.fmf.uni-lj.si/pub/networks/data/map/USpowerGrid.net> |
| *Zewail*[45] | the reference relationship between papers | <http://www.cise.ufl.edu/research/sparse/matrices/Pajek/Zewail.html> |
| Model networks | *WS*[2] | generated by the open source tool *Gephi*’s *BA* Scale free Model B and by setting the number of nodes=200 and the number of edges=400 | |
| *ER*[15] | generated by *Gephi*’s *ER G*(*n, p*) Model and by setting the number of nodes *n*=200 and the probability of edge existence between all pairs of node *p*=0.02 | |
| *BA*[3] | generated by *Gephi*’s WS small world Model Alpha and by setting the number of nodes *n*=200, the average degree of the graph *k*=4 and a tunable parameter=3.5 | |
